# Kératoacanthome géant de la paupière

**DOI:** 10.11604/pamj.2013.16.15.3112

**Published:** 2013-09-13

**Authors:** Zouheir Hafidi, Rajae Daoudi

**Affiliations:** 1 Université Mohammed V Souissi, Service d'Ophtalmologie A de l'hôpital des spécialités, Centre hospitalier universitaire, Rabat, Maroc

**Keywords:** Kératoacanthome, paupière, tumeur, Keratoacanthoma, eyelid, tumor

## Image en médicine

**Figure 1:**
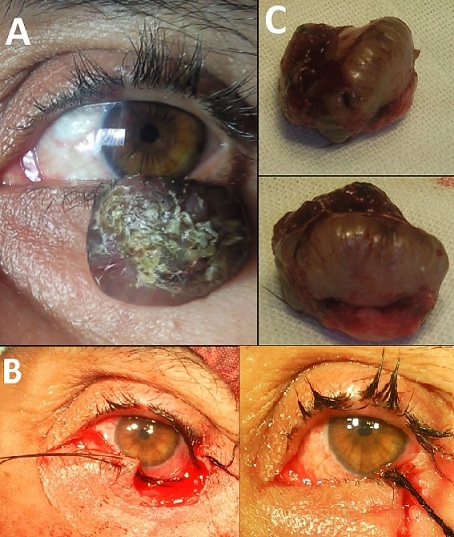
(A) aspect préopératoire montrant une large tumeur de la paupière inférieure avec un bourrelet périphérique à surface lisse et télangectasique entourant un cratère central rempli de matériel corné; (B) aspect préopératoire et post opératoire immédiat; (C) aspect macroscopique de la tumeur après

Il s'agit d'une patiente âgée de 51 ans qui consulte pour une lésion tumorale de la paupière inférieure gauche d’évolution rapide (5 jours), apparue et développée sur une lésion ancienne et stable depuis une dizaine d'années (Figure 1-A). A l'examen, il y avait une lésion bourgeonnante violacée, bien limitée de 3 cm sur 2.5 cm, avec un bourrelet périphérique à surface lisse et télangectasique entourant un cratère central rempli de matériel corné. Faisant évoquer un kératoacanthome. Une biopsie exérèse a été réalisée sous anesthésie locale (Figure 1-B) et l'examen anatomopathologique a confirmé le diagnostic en révélant une tumeur épithéliale nodulaire bien limitée, d'aspect cratériforme, avec des éperons latéraux sans prolifération épidermoïde. L’évolution en postopératoire était favorable, sans aucune récidive après un recul de 3 années. Le principal diagnostic différentiel du kératoacanthome, aussi bien sur le plan clinique qu'histologique, est le carcinome épidermoïde verruqueux dont le pronostic et la prise en charge sont totalement différents. Il est alors impératif de réaliser une biopsie de la totalité de la lésion, portant sur le cratère central et sur le bourrelet périphérique (biopsie transfixiante ou en quartier d'orange) avec une marge de sécurité. L’évolution du kératoacanthome est en général spontanément favorable mais l'exérèse précoce reste le traitement de référence devant le doute diagnostique le risque de récidive et de cicatrice inesthétique surtout en cas de localisation palpébrale car le risque d'exposition cornéenne impose une reconstruction parfois de réalisation difficile.

